# Fabrication of ZnO/CNTs for Application in CO_2_ Sensor at Room Temperature

**DOI:** 10.3390/nano11113087

**Published:** 2021-11-16

**Authors:** Rana Saad, Ahmed Gamal, Mohamed Zayed, Ashour M. Ahmed, Mohamed Shaban, Mohammad BinSabt, Mohamed Rabia, Hany Hamdy

**Affiliations:** 1 Nanophotonics and Applications Laboratory, Physics Department, Faculty of Science, Beni-Suef University, Beni-Suef 62514, Egypt; ranasaad811@gmail.com (R.S.); a_gamal21@yahoo.com (A.G.); m.zayed88ph@yahoo.com (M.Z.); ashour.elshemey@gmail.com (A.M.A.); MOH.RABIE17@yahoo.com (M.R.); hshamdy@hotmail.com (H.H.); 2 Department of Physics, Faculty of Science, Islamic University of Madinah, P.O. Box 170, AlMadinah Almonawara 42351, Saudi Arabia; 3 Chemistry Department, Faculty of Science, Kuwait University, P.O. Box 5969, Safat 13060, Kuwait; Mohammad.binsabt@ku.edu.kw; 4 Polymer Research Laboratory, Chemistry Department, Faculty of Science, Beni-Suef University, Beni-Suef 62511, Egypt

**Keywords:** ZnO/CNTs thin film, CO_2_ gas, sensor response, stability, spray pyrolysis

## Abstract

Thin films of ZnO and ZnO/carbon nanotubes (CNTs) are prepared and used as CO_2_ gas sensors. The spray pyrolysis method was used to prepare both ZnO and ZnO/CNTs films, with CNTs first prepared using the chemical vapor deposition method (CVD). The chemical structure and optical analyses for all the prepared nanomaterials were performed using X-ray diffraction (XRD), Fourier transformer infrared spectroscopy (FTIR), and UV/Vis spectrophotometer devices, respectively. According to the XRD analysis, the crystal sizes of ZnO and ZnO/CNTs were approximately 50.4 and 65.2 nm, respectively. CNTs have average inner and outer diameters of about 3 and 13 nm respectively, according to the transmitted electron microscope (TEM), and a wall thickness of about 5 nm. The detection of CO_2_ is accomplished by passing varying rates of the gas from 30 to 150 sccm over the prepared thin-film electrodes. At 150 sccm, the sensitivities of ZnO and ZnO/CNTs sensors are 6.8% and 22.4%, respectively. The ZnO/CNTs sensor has a very stable sensitivity to CO_2_ gas for 21 days. Moreover, this sensor has a high selectivity to CO_2_ in comparison with other gases, in which the ZnO/CNTs sensor has a higher sensitivity to CO_2_ compared to H_2_ and C_2_H_2_.

## 1. Introduction

Fast industrialization has a number of negative environmental consequences, including global warming, water pollution, climate change, and ocean acidification [1,2]. As a result, eliminating water and air pollution has been a rising scientific issue in recent decades. Different techniques have been introduced for air and water pollutants’ treatment, including plasma-based techniques [3,4,5]. When dealing with air pollution, it is important to first identify harmful gases. Carbon dioxide (CO_2_) is one of the numerous harmful gases that contribute to air pollution. This is stimulating extensive research and collaborative efforts for CO_2_ detection, monitoring, capture, and storage [6,7,8]. Interestingly, CO_2_ monitoring is important not only for air quality but also for health, industry, and agriculture [9,10,11].

Metal oxide semiconductor (MOS) gas sensors are now widely used in many industries and public buildings to detect hazardous and poisonous gases. This is due to the advantages of MOS gas sensors, which include low power consumption, low cost, high sensitivity, and quick response, with excellent microelectronic processing compatibility [12]. To improve the CO_2_ sensor, several semiconductor materials, such as tin dioxide (SnO_2_), tungsten trioxide (WO_3_), copper oxide (CuO), and zinc oxide (ZnO), were used [13,14,15]. ZnO is an n-type semiconductor with a wide optical bandgap energy (3.37 eV). It is also abundant, low in cost, environmentally friendly, and chemically stable. As a result of the quantization effect, size reduction can introduce many novel properties into ZnO nanomaterials. Zinc oxide is, therefore, a multifunctional material, with applications in optoelectronics, electronics, adsorbents, gas sensors, photocatalysts, and solar energy conversion devices [16,17,18,19].

There are many methods for the preparation of ZnO nanomaterials, such as spray pyrolysis, hydrothermal, magnetron sputtering, co-precipitation, electrodeposition, and pulsed laser deposition [20]. Spray pyrolysis is a simple and inexpensive technique to make high-throughput ZnO nanoparticles. However, ZnO has several drawbacks, including low long-term stability, a high working temperature, and a high applied bias voltage, which limit its applications for CO_2_ gas sensors. Kang et al. discussed numerous ways for improving the sensitivity and selectivity of ZnO-based sensors, as well as the likely sensing mechanism of these composites [17]. Padmanathan et al. used magnetron sputtering to create a nanostructured ZnO thin film. The maximum sensitivity was ~1.1% at 1000 ppm of CO_2_ under 300 °C [21]. Waghuley et al. used a screen-printing method to create a ZnO film with a CO_2_ sensitivity of 0.9% at room temperature (RT) [22]. Arun et al. used a screen-printing technique to create an Al-doped ZnO film. The highest sensitivity was observed at 250 °C, with a long recovery time [23].

On the other hand, carbon nanomaterials such as graphene, graphene oxide (GO), and carbon nanotubes (CNTs) have proper properties for the development of low-cost and high-performance gas sensors [24]. The mechanical, chemical, electrical, and thermal properties of carbon nanotubes are exceptional. Due to their large surface area, structural stability, and high conductivity, carbon nanotubes have superior adsorption ability. In recent years, hybrid materials composed of carbon nanotubes (CNTs) and metal oxide semiconductors (MOS) have formed nanocomposites that combine the properties of the parent materials to form a new group of materials with extraordinary gas-sensing properties. As a result, MOS-CNTs nanocomposite exhibits many improved properties that CNTs and MOS alone cannot achieve for gas-sensing applications.

Nguyen et al. demonstrated that SnO_2_/CNTs nanocomposites outperform pristine SnO_2_ or CNTs materials for NH_3_ gas-sensing properties at room temperature [25]. Feifei et al. demonstrated ZnO/CNTs for ethanol vapor detection at 370 °C with good stability and repeatability [26]. Under UV illumination, Mengning et al. demonstrated TiO_2_/CNTs as acetone vapor sensors at low concentrations [27]. ZnO/CNTs nanocomposites have demonstrated high performance in the detection of many gases and vapors, including toluene, ethanol, carbon monoxide, ammonia, sulfur dioxide, H_2_S, and NO_2_ [27,28,29,30,31].

Although ZnO/CNTs nanocomposite is a promising material for gas sensor devices, our literature search revealed that the use of ZnO/CNTs for CO_2_ sensors at RT has not been investigated. As a result, the current study aims to use spray pyrolysis to create ZnO/CNTs nanocomposite films with enhanced CO_2_ sensing properties at room temperature. The effect of long-term stability and selectivity was also studied. In addition, the gas-sensing mechanism was proposed and thoroughly described.

## 2. Experimental Details

### 2.1. Materials

High-purity zinc acetate dihydrate (Zn (CH_3_COO)_2_·2H_2_O, CAS No. 557-34-6), iron nitrate nonahydrate (Fe (NO_3_)_3_, CAS No. 7782-61-8), cobaltous nitrate hexahydrate (Co (NO_3_)_2_·6H_2_O, CAS No. 10026-22-9), and aluminum nitrate nonahydrate (Al (NO_3_)_3_, CAS No.7784-27-2) were obtained from Loba Chemie, Mumbia, India. Sulfuric acid (H_2_SO_4_, 98%), nitric acid (HNO_3_, 69%), ammonium hydroxide (NH₄OH, 30%), and sodium hydroxide (NaOH) were bought from SDFCL, Mumbia, India. Sodium dodecyl sulfate (NaC_12_H_25_SO_4_) was purchased from El-Nasr chemical company, Egypt. High-purity gases (>99%) such as carbon dioxide (CO_2_), hydrogen (H_2_), acetylene (C_2_H_2_), nitrogen (N_2_), and ethylene (C_2_H_4_) were delivered from TAQA gas, Cairo, Egypt.

### 2.2. Fabrication of CNTs

For fabrication of CNTs, 1.0 g of Fe (NO_3_)_3_·9H_2_O, 1.0 g of Co (NO_3_)_2_·6H_2_O, and 3 g of Al (NO_3_)_3_·9H_2_O were dissolved in 100 mL of distilled water for 1 h under stirring at 750 rpm. Many drops of ammonia solution were added until the pH value became 8 to obtain complete precipitation. After that, the solution aged for 2 h at room temperature, and then it was filtrated and washed with distilled water several times. The product was dried at 80 °C and calcined in a Thermolyne™ Premium Large Muffle Furnace (Model F6010, ThermoFisher Scientific, Carlsbad, CA, USA) at 450 °C for 4 h in atmospheric air to remove excess nitrate. The final powder was used as a catalyst for growth of CNTs by the chemical vapor deposition (CVD) technique. This powder was set in a ceramic boat and heated in a tubular electric furnace under flux from acetylene and nitrogen. C_2_H_2_ with a flow rate of 25 sccm was used as a carbon source and N_2_ was used as a carrier gas. The flow rate ratio of C_2_H_2_ to N_2_ was (1:10) *v/v*. The as-prepared CNTs product from CVD was added to H_2_SO_4_:HNO_3_ at a volume ratio of 1:3 in a round bottom flask and refluxed for 6 h in an oil path at 120 °C. Then, the powder was filtered and washed with distilled water and dried at 80 °C for 4 h.

### 2.3. Fabrication of ZnO

Spray pyrolysis is a low-cost and simple technique for producing high-quality metal oxide films on a glass substrate [32]. Sulfuric acid, acetone, and methanol were used to clean the glass substrates under the ultrasonic technique for 30 min, respectively. To make ZnO thin films, a 0.2 M zinc acetate solution dissolved in deionized water was mixed with methanol in a 1:3 ratio. For 5 min, a ZnO precursor solution was sprayed onto a heated glass substrate with dimensions 2.0 × 1.0 × 0.11 cm^3^ at a rate of 5 mL/min. Through a 0.2 mm nozzle, the air was used as the carrier gas. The distance between the nozzle and the glass substrate was set at 40 cm.

### 2.4. Fabrication of ZnO/CNTs

To reduce agglomerates, 3 mg of CNTs were suspended in deionized water with 0.4 g of sodium dodecyl sulfate (SDS) for 6 h using the ultrasonication technique (ultrasonic cleaner with power 100 W, model UD100SH-3.8Q, MTI Corporation, Richmond, VA, USA). For 2 h, a continuous magnetic stirring at 400 rpm was used to mix the ZnO precursor and CNTs solution at room temperature (25 °C). The mixture was sprayed on hot glass substrates to create the ZnO/CNTs thin film, as described above.

### 2.5. Characterization Techniques

The prepared sample was characterized using various analytical tools, and the material morphology was demonstrated using SEM (SEM Auriga Zeiss FIB, ZEISS Microscopy, Munich, Germany). Furthermore, the SEM device includes a unit for energy dispersive X-ray analysis (EDX; Oxford Link ISIS 300, Concord, MA, USA). An X-ray diffractometer (Bruker/Siemens D5000, XRD, Karlsruhe, Germany) was used to confirm the chemical structure. The Shimadzu FTIR-340 Jasco spectrophotometer was used for Fourier Transform Infrared Spectroscopy (FTIR, Shimadzu, Kyoto, Japan) measurements. In addition, for the optical analyses, a double-beam spectrophotometer (Perkin Elmer, Lamba 950 UV/Vis/NIR, PerkinElmer Inc., Waltham, MA, USA) was used.

### 2.6. Gas-Sensing Measurements

A schematic diagram of the system utilized to assess the gas-sensing characteristics is shown in Supplementary Figure S1. This system incorporates the fundamental measurement circuit for commercially available metal-oxide gas sensors. A 1.0 L glass chamber with rubber O-rings on the top has been created for the gas-sensing measurements. Three holes are located on the top: two for gas entry and outflow, and one for electrical signaling. Drop casting was used to create silver interdigitated electrodes on both ends of the sample, i.e., a thin layer of silver was applied on both ends of the sample, which were connected by copper wires to the Keithley measurement-source unit (model 2400). Commercially accessible CO_2_ (99.9%) and air (contains as much as 3% water vapor) cylinders provide the CO_2_ and air gases. CO_2_ gas was pumped into the sensor chamber to stabilize the sensor resistance. While the target and carrier gases were switched on and off each cycle, the sensor output signal (voltage) was recorded using a Keithley 2400 Sourc-emeter (Tektronix, Beaverton, OR, USA), interfaced with a computer. It should be mentioned that during the data collection, the experimental equipment was kept at ambient temperature. Conducting silver paste was used to produce Ohmic connections on both ends, which served as electrodes. After injecting a varied volume of CO_2_ into the chamber, the sensor output voltage was measured.

## 3. Results and Discussion

### 3.1. Characterization of the Fabricated ZnO/CNTs Nanostructured Thin Film

#### 3.1.1. Structural Properties

**Figure 1:**
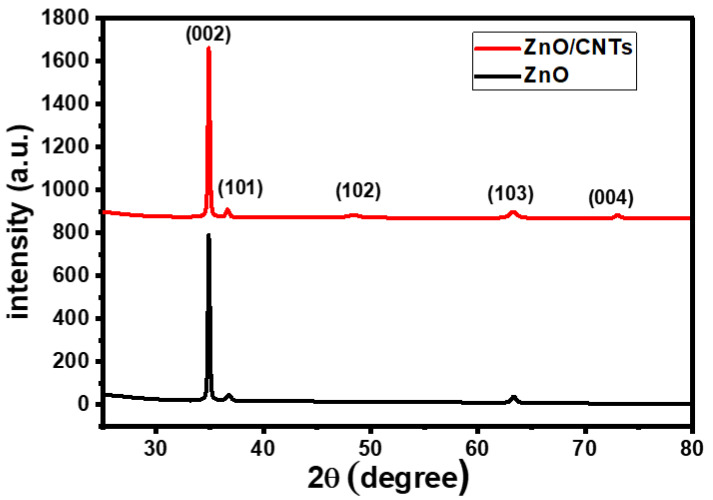
XRD for ZnO and ZnO/CNTs films.

XRD analysis has been performed to confirm the crystal structure and for fabricated thin films. Figure 1 shows the XRD charts of ZnO and ZnO/CNTs nanostructured films deposited on a glass substrate by the spray pyrolysis technique. The crystallographic parameters for XRD patterns obtained from X’Pert software (X’Pert HighScore Plus 3.0.5, Malvern Panalytical Ltd, Cambridge, United Kingdom) analysis are introduced in Table 1. Pure ZnO nanostructures exhibit polycrystalline forms, according to the standard card CDs-89-0510 [33,34,35]. ZnO crystallizes in the hexagonal unit cell with P63mc space group and C6v (6 mm) point group symmetries and has a Wurtzite structure phase. ZnO thin film has a sharp diffraction peak (002) at 2θ = 34.41° and grows preferably perpendicular to the glass surface through the c-axis. According to Miller’s indexes, (101), (102), (103), and (004) planes of ZnO, there are other minor peaks detected at 2θ = 36.68°, 48.49°, 63.31°, and 73.12°. Furthermore, the XRD pattern does not reveal any additional impurity phases for Zn compounds or Zn metal. This demonstrates the use of spray pyrolysis to create pure crystalline ZnO thin films.

Adding CNTs to ZnO changed the structural XRD parameters but not the phase structure of the ZnO film. When ZnO/CNTs are used, the intensity of the main peak (002) increases when compared to pure ZnO. In addition, as shown in Table 1, the line broadening at half the maximum intensity (β) for this peak is narrow. Simultaneously, the peak position was shifted to a larger angle due to induced structural lattice deformation in the ZnO crystal. This suggests that adding CNTs improves the crystallinity of ZnO thin films. Furthermore, no CNT peaks appear due to the low CNT content of the composite and the high intensity of the background [36,37].

The inter-planar distance (d) for (002) planes was increased as presented in Table 1 due to the diffraction angle (2θ) being inversely related to the inter-planar distance according to Bragg’s equation:

(1) nλ=2 d sinθ

where λ is the incident wavelength of the X-ray (= 0.154 nm), d is the inter-planar cell distance, n is the order of diffraction, and θ is the diffraction angle. The crystal size (D) of the ZnO and ZnO/CNTs thin films was estimated by using the Debye-Scherrer equation:

D = 0.9 λ/β cosθ(2)

The crystal size of pure ZnO increased from 17.7 to 27.6 nm after ZnO/CNTs composite formation for the main peak (002) due to the diffraction peak broadening of the ZnO film, as illustrated in Table 1. The average values of the crystallite size based on XRD data were 17.7 nm and 27.6 nm for pure ZnO and ZnO/CNTs composites, respectively. Increasing the particle size of ZnO with the addition of CNTs was observed in previous work [38]. The crystal sizes play an important role in the optical properties of the films.

The lattice parameters (a and c), the unit cell volume (V), the internal parameter (u), the Zn-O bond length (L), the stress (σ), and the dislocation density (δ) of the fabricated ZnO and ZnO/CNTs films were calculated by using the following equations [39,40,41,42]:

(3) $$d_{\left(h\;k\;l\right)}={\left[\frac{4}{3}\left(\frac{h^{2}+k^{2}+h\;k}{a^{2}}\right)+\frac{l^{2}}{c^{2}}\right]}^{-0.5}$$

(4) $$V=\frac{\sqrt{3}}{2}{\;a}^{2}\;c$$

(5) $$u=\frac{a^{2}}{3c^{2}}+\frac{1}{4}$$

(6) $$L=\sqrt{\left(\;\frac{a^{2}}{3}+{\left(0.5-u\right)}^{2}c^{2}\right)}$$

(7) $$\sigma =4.5\times 10^{11}\;\frac{c_{o}-c}{c_{o}}$$

#### 3.1.2. Morphological Analysis

**Figure 2:**
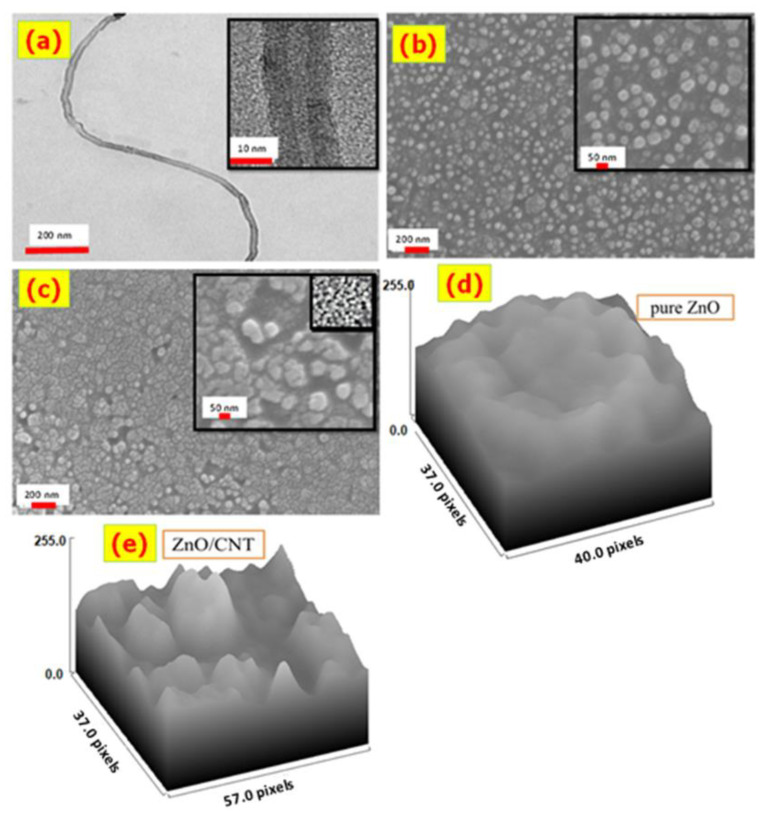
(**a**) HRTEM image of the CNTs. The HRSEM image of the (**b**) ZnO and (**c**) ZnO/CNTs thin films, and (**d**) surface roughness of ZnO and (**e**) surface roughness of ZnO/CNTs.

The HRTEM image of the CNTs prepared in homemade CVD is shown in Figure 2a. This figure clearly shows that CNTs are hollow and tubular structures with a high aspect ratio. The formed CNTs have a length of several micrometers. The average inner and outer diameters of CNTs are about 3 and 13 nm respectively, with a wall thickness of about 5 nm. The high-magnification image for CNTs in Figure 2 shows an aligned lamellae pattern with a lattice spacing of 0.25 nm.

At various magnifications, HRSEM images were used to examine the surface morphology of ZnO and ZnO/CNTs nanostructured thin films. Figure 2b demonstrates that ZnO contains a large number of nanoparticles with nano-spherical shapes. The average diameter of the ZnO nano-spherical particles is about 50.4 nm. As seen in the high-magnification image inserted in Figure 2b, these ZnO nanoparticles self-assemble to create a large space between them.

Figure 2c demonstrates that integrating CNTs results in multi-shape nanoclusters on the surface of the deposited film. The deposited ZnO/CNT nanoparticles coalesced and were intergrown with one another to produce a continuous layer that nearly entirely covered the glass substrate. The nanoclusters have an average size of 65.2 nm, i.e., the incorporation of CNTs increases the average particle size from 50.4 to 65.2 nm. The ZnO/CNTs film has a rougher surface and a larger surface area to volume ratio than the ZnO film due to the uneven distribution of nanoclusters of diverse irregular sizes and shapes.

Additionally, the high-magnification HRSEM image inserted in Figure 2c shows very small nanopores. These nanopores are distributed non-uniformly with varying diameters. The nanopores’ structure shows a high possibility for gas adsorption on the film surface, and this allows increasing the sensitivity of the ZnO/CNTs film at room temperature [43,44,45]. Figure 2d,e shows the surface roughness of ZnO/CNTs and ZnO analyzed by ImageJ software. The surface roughness of ZnO/CNTs is higher than that of ZnO, which increases the surface area and gas sensitivity.

#### 3.1.3. Chemical Composition

**Figure 3:**
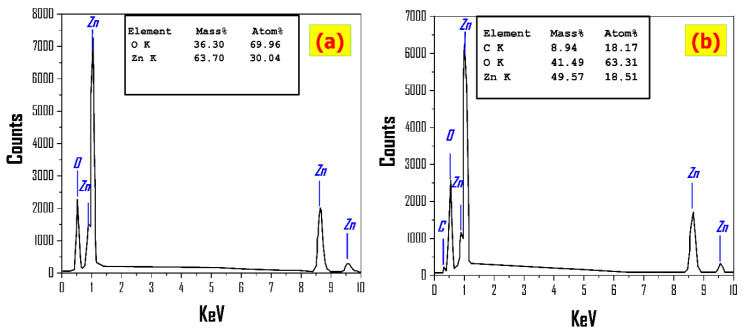
EDX patterns for (**a**) pure ZnO and (**b**) ZnO/CNTs, with the inset showing the chemical composition ratio.

The energy dispersive X-ray (EDX) technique is a promising low-cost, quick, and non-destructive tool for identifying quantitative elemental information and its ratio in a sample. The EDX spectrum of pure ZnO nanostructures and ZnO/CNTs is shown in Figure 3. The figure’s inset table provides quantitative information on the chemical composition ratio of samples.

The EDX chart of ZnO film in Figure 3a confirms the presence of Zn and O signals with mass% of 63.70% and 36.30%, respectively. This illustrates the high purity of the sprayed ZnO thin film, which is consistent with the XRD results. The atomic ratios of Zn and O were found to differ from the ZnO stoichiometric ratio. This is due to the fact that the oxygen signal is received from the ZnO nanostructured film and the glass substrate as a result of the thicknesses of the thin film being smaller than the EDX interaction volume (≥1 cube μm^3^). The EDX chart in Figure 3b certifies the presence of Zn, C, and O peaks, and the mass ratio quantitative analysis for ZnO/CNTs film is 49.57%, 8.94%, and 41.49% for Zn, C, and O, respectively. This indicates CNTs incorporated with the fabricated ZnO thin films.

#### 3.1.4. Fourier Transform Infrared Spectroscopy (FTIR)

FTIR spectroscopy is a technique for obtaining an infrared spectrum of absorption or emission of a solid, liquid, or gas [46]. An FTIR spectrometer simultaneously collects high-resolution spectral data over a wide spectral range. FTIR spectroscopy is used to quickly and definitively detect chemicals. It can be used in all stages of the product lifecycle, including design, manufacturing, and failure analysis [47,48].

**Figure 4:**
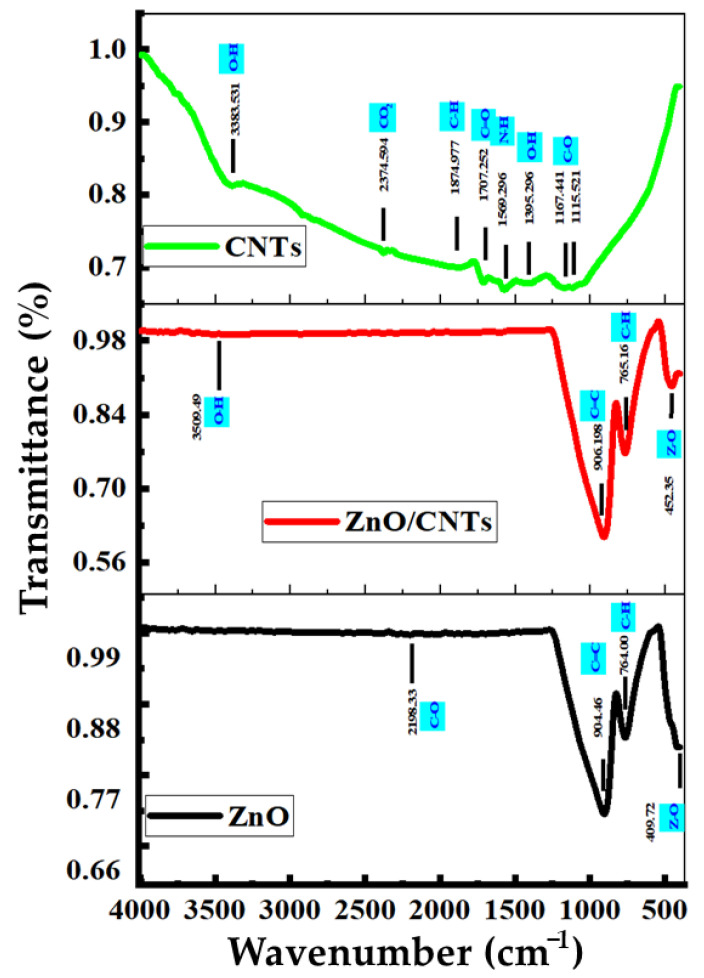
FTIR spectra for ZnO, CNTs, and ZnO/CNTs samples.

The fundamental concept at work is that different elements’ bonds absorb light at different frequencies. An infrared spectrometer is used to measure the light, resulting in an infrared spectrum [49,50]. FTIR spectroscopy was performed to further understand the formation of ZnO, CNTs, and ZnO/CNTs thin films and the surface chemistry of the prepared nanostructures [51]. Figure 4 shows the FTIR spectrum of the ZnO, CNTs, and ZnO/CNTs in the range of 4000–400 cm^−1^ wave number. For ZnO nanomaterial, the band at 3509.49 cm^−1^ is related to the OH group from the ZnO stretching vibration due to adsorbed H_2_O molecules on the ZnO surface. The band at 904 cm^−1^ is related to the C-O bond formed due to using methanol as a solvent during deposition of ZnO nanomaterial. This band appears in ZnO/CNTs at about 906 cm^−1^, related to the C-O function group in CNTs due to the formation of little OH groups in the surface of CNTs coming from the preparation procedures.

The peak at 2198 cm^−1^ appears after CO adsorption on the surface of the samples. There is a broadband with very low intensity at 3509.49 cm^−1^ corresponding to the vibration mode of water’s OH group, which indicates the presence of a small amount of water adsorbed on the ZnO/CNTs nanocrystal surface. Additionally, the methanol C–H groups have a band at 755 cm^−1^ for ZnO, and the same band appears at 763.9 cm^−1^ for ZnO/CNTs. In CNTs, the medium band at 3383.53 cm^−1^ exhibits the stretching of O-H, and the transmitted weak band at 1874.97 cm^−1^ is related to C-H bending. In the low-frequency or fingerprint region, a strong band at 415 cm^−1^ is attributed to the Zn-O stretching band, and the band at 452.34 cm^−1^ in ZnO/CNTs is related to the Zn-O bonds on the surface of the CNTs. This peak supports the fact that ZnO nanoparticles are synthesized and attached to the surfaces of CNTs.

Two methods were used to prepare ZnO and ZnO/CNTs with various ratios of oxygen functional groups, such as O-H and C-O, to study their effects on the CO_2_ sensing properties at room temperature. It was found that the percentages of oxygen-containing species, such as –OH in the ZnO/CNTs thin film, significantly affected the sensitivity of ZnO/CNTs thin film to CO_2_ at room temperature. The –OH species can promote sensitivity and also recovery ability concerning CO_2_. This may be because –OH has a greater attraction to CO_2_ and obtains electrons from it, whereas the separation of CO_2_ from –OH is also slightly easier, enabling a better recovery compared to other oxygen functional groups such as C-O [51,52].

#### 3.1.5. Optical Properties

**Figure 5:**
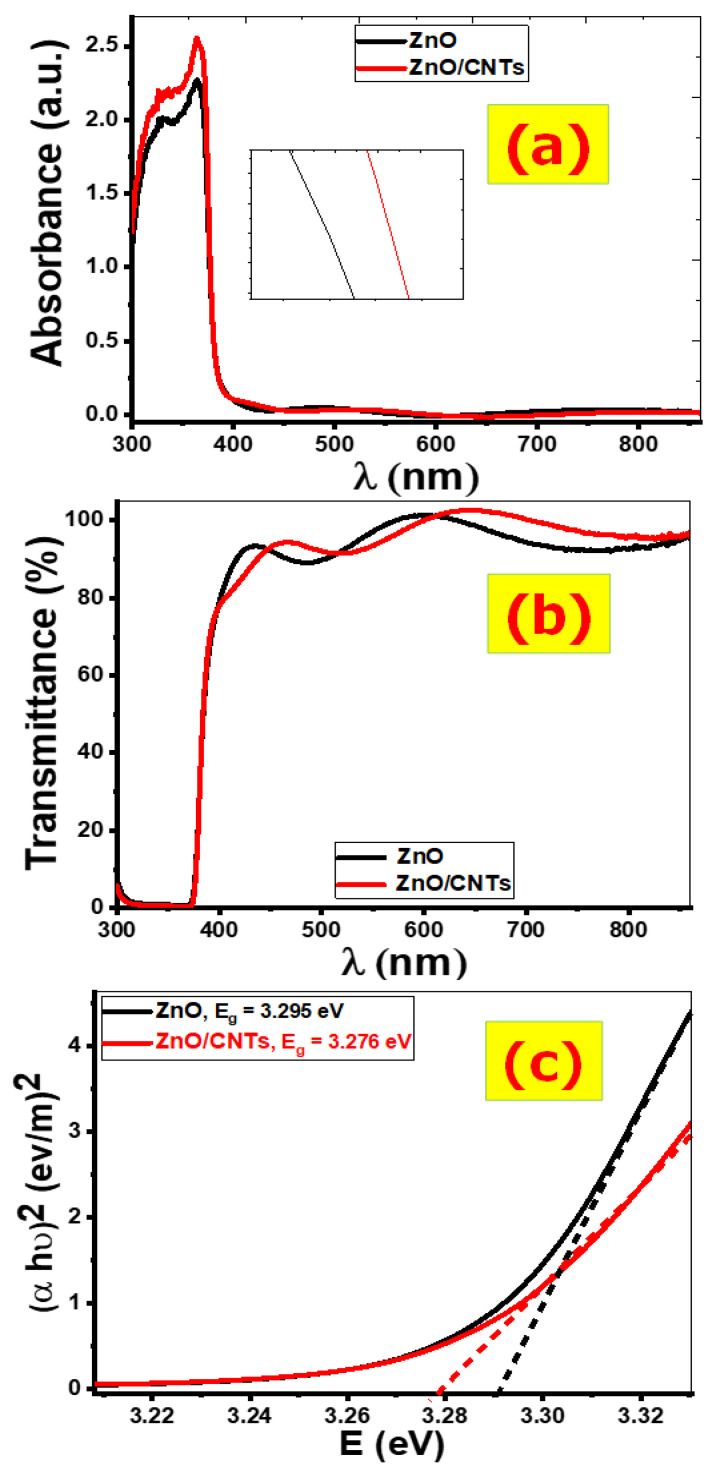
Optical spectra (**a**) absorption, (**b**) transmission, and (**c**) Tauc plots for ZnO and ZnO/CNTs thin films.

Nanomaterials’ optical properties are linked to their electronic properties, such as bandgap energy. Figure 5 shows the absorbance and transmittance spectra from 250 to 900 nm that were studied to reveal the optical properties of ZnO and ZnO/CNTs films. It has been discovered that ZnO and ZnO/CNTs have a strong absorption band in the 300–400 nm range. This demonstrates the high photo-response of ZnO and ZnO/CNTs in this range. The strong light absorption is caused by electronic jumps from the valence band to the conduction band. As shown in Figure 5a, the absorption spectrum of ZnO and ZnO/CNTs decreases sharply after 400 nm and approaches zero for longer wavelengths.

All of the deposited films have a sharp absorption edge, which confirms the material’s crystalline nature. When compared to pure ZnO, the absorption edge of ZnO/CNTs is red-shifted to a longer wavelength region, indicating a decrease in the bandgap value of ZnO/CNTs. Figure 5a shows that the absorbance of ZnO/CNTs films increases, particularly in the UV region. This demonstrates that the presence of CNTs influences the optical properties of ZnO thin films.

The transmittance of the ZnO and ZnO/CNTs films below 400 nm is nearly zero (Figure 5b) because of their high absorption properties in this region. All films exhibit high transmission of nearly 90% above 400 nm. The highest transmittance corresponds to the formation of uniform and smooth surfaces for ZnO and ZnO/CNTs thin films. The transmission curve shows strong fringes due to interference of ZnO and ZnO/CNTs thin films with good optical homogeneity and crystalline structure.

The bandgap energy (Eg) of crystalline semiconductor materials could be estimated by using the Tauc relation (1) [26]:

(8) $${\left(\alpha h\nu \right)}^{2}=q\;\left(h\nu -\;\mathrm{Eg}\right)$$

where α is the absorption coefficient, hν is the photon energy, q is a constant, h is Planck’s constant, and Eg is the direct bandgap between the conduction (C.B) and valence bands (V.B). Therefore, when plotting ${\left(\alpha \;h\;\nu \right)}^{2}$ versus hν, the extrapolating straight line to the hν axis intercept gives the bandgap energy transition value of all the materials (Eg=h ν at α=0), as shown in Figure 5c. The resultant band gaps of ZnO and ZnO/CNTs were 3.295 and 3.276 eV respectively, as shown in Figure 5c. The decreasing Eg of ZnO/CNTs, due to the increase in the crystallite size of the main peak, occurs according to the quantum confinement effect [53].

### 3.2. Gas-Sensing Measurements

**Figure 6:**
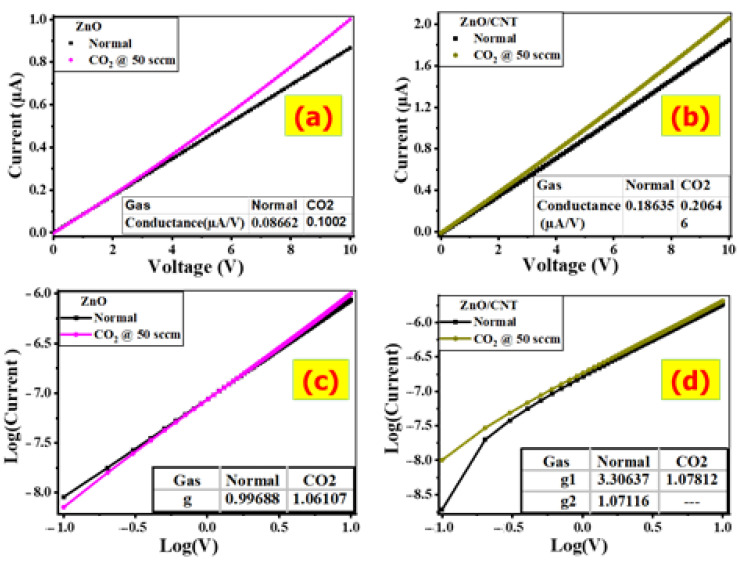
I-V and Log (I)-Log (V) for (**a**,**c**) ZnO and (**b**,**d**) ZnO/CNTs films.

The ability of the ZnO and ZnO/CNTs films for CO_2_ detection was experienced in a homemade gas chamber system at room temperature (25 °C) under the air environment. Gas sensing was measured by observing the change in the resistance of films between two electrodes under various CO_2_ concentrations. The concentration of gases proportionally affects the resistance change of films [54]. The proposed sensors’ operation is based on the charge/electron exchange between CO_2_ molecules and the surface of films. The characteristic current versus voltage (I-V) curve can provide information about gas–film contacts. Figure 6a,b depicts the I-V curves of ZnO and ZnO/CNTs sensors in air and CO_2_ environments with a constant flow rate of 50 sccm (standard cubic centimeters per minute) and bias voltage ranges ranging from 0 to 10 V, respectively. For I-V curves in the air and CO_2_ environments, both films exhibit approximately linear Ohmic behavior. The Ohmic behavior is critical to sensing properties [55].

When both films are subjected to the same voltage, the flowing current increases under 50 sccm of CO_2_ molecules when compared to the air environment. When ZnO and ZnO/CNTs films are exposed to CO_2_ gas, their resistance decreases. This can be attributed to the oxidizing nature of CO_2_ gas, which causes the resistance of the n-type material to decrease. As a result, the concentration of CO_2_ gas can be determined using the resistance values of ZnO and ZnO/CNTs films. In CO_2_, the current intensity of ZnO/CNTs becomes two-fold when compared to ZnO at the same voltage. This means that the CNTs enhanced the conductivity and hence the value of the electric current.

Based on the I-V curve, the resistance (R) and dc conductance (G_dc_) values for films can be calculated by using the curve slopes. For ZnO, the resistance increases from 9.98 MΩ in CO_2_ to 11.54 MΩ in air. Under the applied 50 sccm of CO_2_, the conductance increases to 0.1002 and 0.20646 µS for ZnO and ZnO/CNTs, respectively. The dc conductance (G_dc_) of ZnO and ZnO/CNTs increases in the CO_2_ environment because a smaller number of carriers is trapped in the boundaries of the grains and more electrons are liberated.

The changes in the value of resistance/conductance demonstrate the effect of gas adsorption on the films and their ability to detect CO_2_ gas presence. For ZnO/CNTs, the conductance is greater than two times compared to ZnO in the CO_2_/air environment due to the high conductivity of CNTs nanomaterials, and hence the easiest of electron transport finally enhances the electric conductivity of ZnO/CNTs. This indicates that ZnO/CNTs film is a more efficient CO_2_ sensor than ZnO film.

The plot of log (V) against log (I) for ZnO and ZnO/CNTs films in different environments is illustrated in Figure 6c,d, respectively. This figure can be used to estimate the values of the nonlinear coefficient (g) based on the empirical formula I = C V^g^, where C is a constant. Then, the nonlinearity coefficient is determined by the relation g = d[log (I)]/d[log (V)]. The g parameter is used to measure the degree of nonlinearity of an element as a function of the applied voltage. The figure of Log (V) and Log (I) has one stage for ZnO with g = 0.996 in air. ZnO/CNTs have two stages and hence two values of g (g_1_ = 3.306 and g_2_ = 1.071) in air. This indicates the non-ohmic I-V behaviors of the ZnO/CNTs film in the air. Smaller grain sizes can increase the nonlinearity coefficient because the number of grain boundaries per unit thickness increases [56].

### 3.3. Dynamic Response

**Figure 7:**
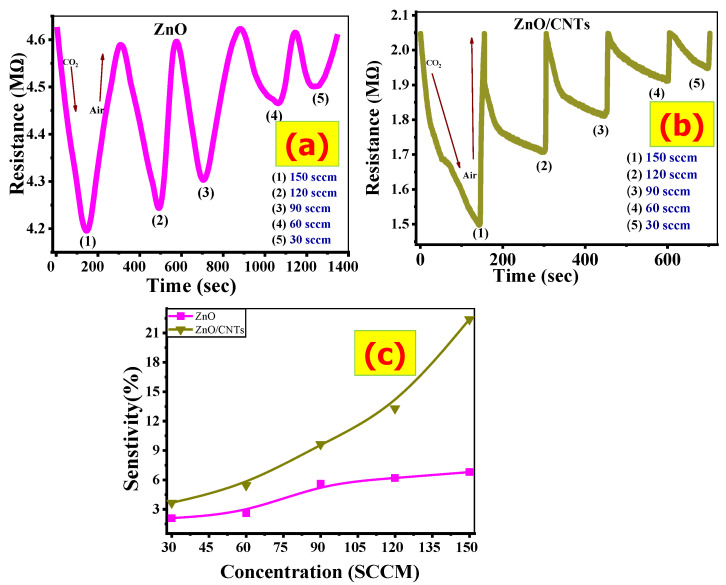
(**a**,**b**) Dynamic response of ZnO and ZnO/CNTs films, and (**c**) sensitivity.

Transient response measurements were carried out to better understand the sensing properties of the ZnO and ZnO/CNTs sensors towards CO_2_ concentrations. Figure 7a,b depicts the dynamic response of ZnO and ZnO/CNTs to CO_2_ at different concentrations at room temperature (30, 60, 90, 120, 150 sccm). These behaviors reflect the interaction of CO_2_ molecules with gas molecules on the film’s surface via adsorption/desorption processes. With the insertion of CO_2_ molecules, the sensor’s resistance (R_CO2_) decreases with the detection time until it reaches a stable stage. This reflects the behavior of an n-type semiconductor (the charge carriers are electrons).

When the CO_2_ gas stream is interrupted and the air is injected into the chamber system, the resistance of the sensor in the air (R_air_) value rapidly increases to achieve the baseline resistance. This demonstrates an excellent recovery sensor. This type of behavior for ZnO as a CO_2_ gas sensor has been discussed by several research groups [21,57,58,59]. The minimum value of resistance decreases with an increase in the CO_2_ concentrations from 30 to 150 sccm for both ZnO and ZnO/CNTs films. This behavior is due to the fact that the increase in the CO_2_ environment leads to more electrons being liberated and the resistance decreases.

For practical sensors, sensor sensitivity is a very vital factor. This parameter can be calculated from Figure 7a,b. The sensor response (sensitivity, S%) is calculated based on:

(9) $$S\%=\left|\frac{R_{{\mathrm{CO}}_{2}}-{\;R}_{\mathrm{air}}}{{\;R}_{\mathrm{air}}}\right|\times 100$$

where $R_{{\mathrm{CO}}_{2}}$ and $R_{\mathrm{air}}$ refer to the measured resistance in CO_2_ and air environments. The value of $R_{{\mathrm{CO}}_{2}}$ is obtained from the dynamic response after a certain exposure time (1 min) to CO_2_, and $R_{\mathrm{air}}$ is measured in the same conditions. Figure 7c shows the sensor responses versus CO_2_ concentration for ZnO and ZnO/CNTs films, respectively. For both materials, the sensor response is increasing with the CO_2_ gas concentration. The ZnO/CNTs film displays a very high sensor response relative to that of the ZnO film. ZnO has high electrical resistance at room/low temperature. This leads to a negative effect on the surface reactions between ZnO and CO_2_ gas, which are slow at room temperature, leading usually to a low sensitivity [60].

At a low CO_2_ concentration, the response is low because only a small amount of CO_2_ interacts with the active sites on the ZnO/CNTs surface. By increasing the CO_2_ concentration, the response proportionally increases due to more CO_2_ gas molecules adsorbing on the surface, leading to a higher sensing response. This agrees with many previous works [61,62].

The sensor response gap between the ZnO/CNTs and ZnO films is small at a low concentration of CO_2_. However, at high CO_2_ concentrations, the gap becomes much larger. As the CO_2_ concentration is raised from 30 to 150 sccm, the S% of the ZnO film is semi-linearly increased from 2.1% to 6.8%, while the S% of the ZnO/CNTs film is sharply increased from 3.6% to 22.4%. The pores in the ZnO/CNTS film led to an increase in the surface area and improvement in the sensitivity to CO_2_ gas. The sensor response of the ZnO/CNTs sensor is more than three times higher than that of the ZnO sensor at 150 sccm CO_2_.

### 3.4. Response and Recovery Time

**Figure 8:**
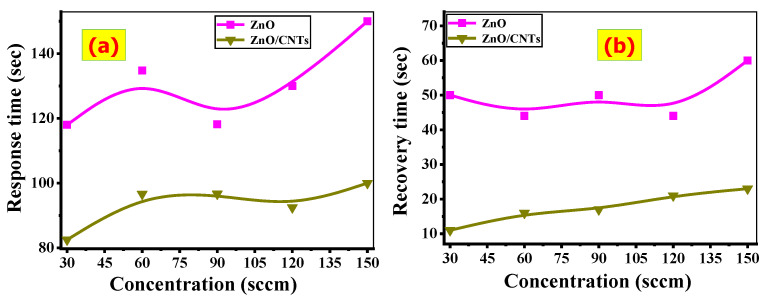
(**a**) Response and (**b**) recovery times for ZnO and ZnO/CNTs films.

The response time (t_resp_) is the time required to achieve 90% of the full resistance variation after inserting CO_2_ into the chamber system. The recovery time (t_recov_) is the amount of time required to achieve 90% of the full resistance variation after inserting air into the chamber system. Figure 8a,b depicts the response/recovery times obtained from ZnO and ZnO/CNTs films at various CO_2_ concentrations. The t_resp_ of the ZnO/CNTs film is lower than that of the ZnO film. At 30 sccm of CO_2_, the response time for the ZnO/CNTs film is approximately 82.5 s, and for the ZnO film is approximately 118 s. The t_recov_ of the ZnO/CNTs film is lower than that of the ZnO film. It is observed that the recovery time at 150 sccm of CO_2_ is about 67 and 23 s for ZnO and ZnO/CNTs films, respectively.

The detection limit (DL) is defined as the lower concentration at which the sensor can detect the gas. It can be calculated from the standard deviation (SD) of the sensor response at the low concentration and the slope of the straight segment at low concentrations, as follows:

DL = 3 × SD/Slope (10)

DL for ZnO and ZnO/CNTs is calculated to be 5.5 and 2.1 sccm, respectively. Signal to noise ratio (S/R) can be calculated using the full width at half maximum of the response peak (Δt_FWHM_) and the resonance response time (t_resonance_), as follows: S/R = t_resonance_/Δt_FWHM_. The average noise level is 9.29 and 2.19 for ZnO and ZnO/CNTs films in a CO_2_ environment, respectively.

### 3.5. Repeatability (Reducibility) and Stability

**Figure 9:**
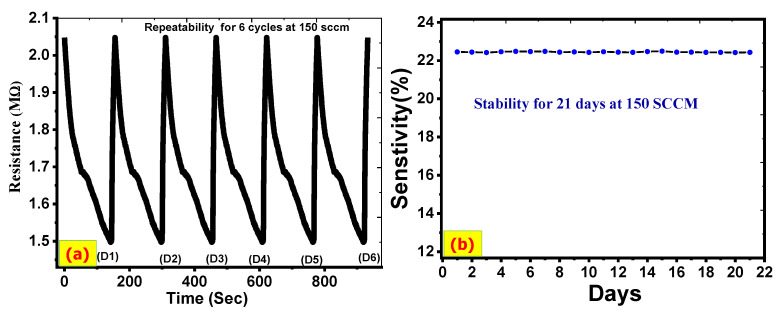
(**a**) Repeatability and (**b**) stability for ZnO/CNTs at 150 sccm CO_2_.

Repeatability and stability are critical performance indices for the sensor’s long-term operation. Six continuous dynamic CO_2_ response cycles with the same concentration (150 sccm) at room temperature were studied to test the repeatability of the ZnO/CNTs sensor. As shown in Figure 9a, the six cycles are very close, with only a slight oscillation of electrical resistance. In addition, the ZnO/CNTs sensor recovered quickly to its initial baseline. Stability studies a sensor’s ability to maintain a reproducible response performance over time in an identical environment.

No solutions have come into contact with the gas sensor electrode. For 21 days, the stability of the ZnO/CNTs electrode for a CO_2_ gas sensor was tested. The outcome was extremely similar to the previous ones, as shown in Figure 9b. This period is ideal for testing, especially for gas sensor electrodes that operate at room temperature and are not exposed to any solutions. Additionally, the ZnO/CNTs sensor has chemical and thermal stability. This agrees well with previous works, whereas the stability of the gas sensor was measured for almost 21 days [63,64,65].

The coefficient of variation (CV) examines the reproducibility of the gas sensor [66]. It is the ratio of standard deviation (SD) to the mean of response magnitude for a repeated response (µ) of the sensor at constant concentration towards a certain gas: CV = SD/µ. The value of CV = 0.005 for the ZnO/CNTs film. This designates that the ZnO/CNTs sensor has good reproducibility and can be reused many times with excellent performance.

The sensing material does not suffer essential changes in the structural and electrical properties. The high stability of the ZnO/CNTs sensor is due to its good ability to adsorb/desorb the CO_2_ molecules on its surface that have a lot of active sites. Hence, the ZnO/CNTs sensor exhibits good stability and repeatability, which makes it very important for commercial applications.

### 3.6. ZnO/CNTs Selectivity to CO_2_ Gas

Selectivity is an important factor in determining the gas-sensing properties. Even though ZnO/CNTs hybrid sensors have a better response to CO_2_, selectivity is a major issue for metal oxide-based gas sensors. Typically, the selectivity of sensors is investigated by alternating the exposure of various gases on the tested devices and determining the sensor response ratio for individual gases:

(11) $$\mathrm{Selectivity}\;\left(\%\right)=\frac{S_{\mathrm{other}\;\mathrm{gas}}}{{\;S}_{\mathrm{target}\;\mathrm{gas}}}\times 100$$

**Figure 10:**
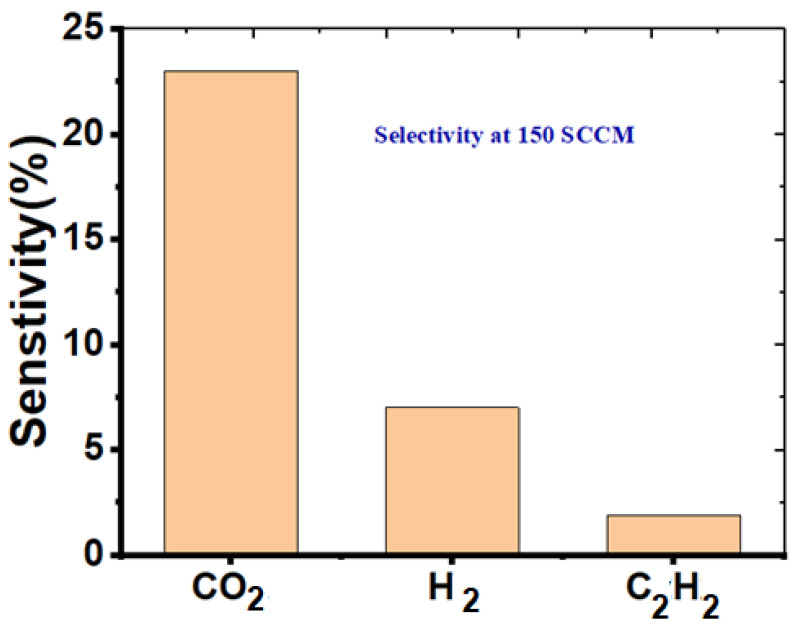
The selectivity of ZnO/CNTs for CO_2_ gas.

Therefore, the sensor response of the ZnO/CNTs was tested by exposure to various gases (CO_2_, H_2_, and C_2_H_2_) successively with the concentration of 150 sccm at room temperature. The sensor’s response to CO_2_ was higher than other tested gases (S_CO2_ > S_H2_ > S_C2H2_), as seen in Figure 10.

The selectivity factor is the ratio between its sensitivity to the target gas molecule and its sensitivity to another interfering gas molecule. The calculated results for devices based on ZnO/CNTs at 150 sccm were S_CO2_/S_H2_ = 3.1% and S_CO2_/S_C2H2_ = 11.9%. The excellent selectivity of ZnO/CNTs is due to the CO_2_ molecule being more likely to be absorbed by ZnO/CNTs than other gases. Hence, the reaction between the CO_2_ molecule and the oxygen ions on the surface more easily occurs, which shows a higher sensing response to CO_2_ than other gases. This demonstrates an excellent selectivity to CO_2_ of the ZnO/CNTs samples at room temperature in a complex environment with different kinds of gases.

### 3.7. Gas-Sensing Mechanism

In general, the CO_2_ gas-sensing mechanism of the ZnO and ZnO/CNTs films is expressed as the change of electrical resistance with the adsorption/desorption of gaseous molecules. ZnO is an n-type semiconductor that contains free electrons. When the ZnO sensor is exposed to air, its surfaces will adsorb the surrounding oxygen molecules. The adsorbed oxygen molecules react with the ZnO surface, where they act as electron acceptors. The oxygen molecules partially extract free electrons from the conduction band of ZnO, leaving the oxide surface partially positively charged and forming oxygen ions partially negative charged (O^2−^), or similar charges, in which the reaction can be written as follows:

O_2_ (gas) → O_2_ (ads) (12)

O_2_ (ads) + e^−^ → O_2_^–^ (ads) (13)

O_2_^–^(ads) + e^−^ → 2O^–^ (ads) (14)

O^–^ (ads) + e^−^ → O^2–^ (ads) (15)

The electron depletion layer is produced on the ZnO surface. Therefore, the resistance of the sensor increased (low conductivity) and the reduction of the conductive cross-sectional area occurred. Adding CNTs into ZnO affects the morphology and the electrical conduction for ZnO/CNTs, which in turn plays a role in the sensor response. The improvement of the CO_2_ sensor response of the ZnO/CNTs compared with ZnO is due to many reasons, as described below.

Firstly, the ZnO/CNTs composite has a higher specific surface area than ZnO due to the tubular structure of CNTs and the porous features of the nanoclusters. Hence, the ZnO/CNTs surface can offer many pathways for diffusing the CO_2_ gas molecules into the interior of the ZnO/CNTs film. The high surface area provides abundant active sites to absorb more target molecules of gas and hence enhance the response of the sensor [67].

Secondly, the high conductivity of the CNTs enhances the electron transport velocity in the ZnO/CNTs film [26]. Therefore, the CNTs can be serving as highly conductive channels or networks for electrons between ZnO-ZnO nanoparticles and ZnO-CO_2_. The fast charge transfer improves the performance of the sensor [30].

Thirdly, the CNTs have very high thermal conductivity, thus effectively improving the thermal conductivity of the ZnO/CNTs carrier material [68]. The high thermal conductivity accelerates the desorption of gas molecules, which improves the response time and stability of the sensor.

Fourthly, the p-n heterojunction is formed at the interface between ZnO and CNTs due to ZnO and CNTs being n-type and p-type semiconductors, respectively [69]. The difference in work functions of CNTs and ZnO is relatively small, and leads to the formation of Ohmic contacts between ZnO and CNTs. The electrons will transfer from the conduction band of ZnO to CNTs and holes will flow from CNTs to ZnO until this creates the in-built electric field between the n-ZnO/p-CNTs heterojunction [70,71]. When the CO_2_ interacts with ZnO/CNTs surface, the field can be decreased owing to the easy flow of the charge carriers, which leads to a much higher sensing response [30,72]. The potential barrier between the n-ZnO/p-CNTs heterojunction is decreased under exposure to CO_2_ gas, which offers an easy flow of the charge carriers and hence a higher sensing response [30,71,72].

Table 2 illustrates the performance of many nanomaterials for the detection of CO_2_ gas [73,74,75,76]. As shown in this table, the response of the presented sensor is much higher than the values for the sensors previously reported in the literature.

## 4. Conclusions

In this paper, ZnO and ZnO/CNTs thin-film electrodes were prepared and used as gas sensors for CO_2_ detection. As electrodes for CO_2_ gas sensing, ZnO and ZnO/CNTs were used. XRD, FTIR, SEM, and TEM analyses were used to determine the chemical structure and morphology of the prepared nanomaterials. The nanoparticle sizes of the prepared materials were approximately 50.4 and 65.2 nm for ZnO and ZnO/CNTs, respectively. After traces of CNTs were composited with ZnO, the sensor responsivity was increased to 22.4%, compared to 6.8% for bare ZnO. This sensor has excellent CO_2_ detection stability over a period of more than 21 days. Additionally, the sensor has a higher selectivity for CO_2_ than other gases, such as H_2_ or C_2_H_2_.

## Figures and Tables

**Table 1:** The XRD parameters for the ZnO and ZnO/CNTs thin films for the (002) plane.

Parameters	ZnO	ZnO/CNTs
Position (2θ°)	34.9024	34.8877
d-spacing (Å)	2.5707	2.57175
Relative Intensity (%)	100	100
Crystallite size (nm)	17.7	27.6
Micro-strain (%)	0.197346	0.255058
(a = b) (Å)	3.22271	3.20763
c (Å)	5.1435	5.1414
V (Å^3^)	46.263	45.786
U	0.38086	0.37974
L (Å)	1.95895	1.95241
σ × 10^−6^	2.35537	3.93763

**Table 2:** Performance parameters for many nanomaterials for detection of CO_2_ gas reported in this review and previous studies.

Materials	Response	Ref.
Graphene/PEI/PEG	11.5	[73]
Oxide-polymer La_2_O_2_CO_3_/P[VBTMA][PF6]	1.12	[74]
Graphene/Y_2_O_3_	1.08	[75]
SnO_2_/rGO	0.07	[76]
rGO	0.179	[76]
ZnO/CNTs	22.4	This Work

## Data Availability

The data presented in this study are available on request from the corresponding author.

## Supplementary Materials

The following are available online at https://www.mdpi.com/article/10.3390/nano11113087/s1, Figure S1: Schematic diagram for the homemade CO_2_ gas sensor testing system.
