# Supplementary material for: Fabrication of ZnO/CNTs for Application in CO2 Sensor at Room Temperature
Source: Nanomaterials (Basel). 2021 Nov 16;11(11):3087. doi: 10.3390/nano11113087 (PMC8624847; doi:10.3390/nano11113087)
Supplement: Supplementary file 1 [file nanomaterials-11-03087-s001.zip › nanomaterials-1415134-supplementary.pdf]

Supplementary data

Fabrication of ZnO/CNTs for application in CO_2 sensor at room temperature

Rana Saad[1], Ahmed Gamal[1], Mohamed Zayed[1], Ashour M. Ahmed[1], Mohamed Shaban[1,2*], Mohammad BinSabt[3], Mohamed Rabia[1,4], Hany Hamdy[1]

[1] Nanophotonics and Applications Lab, Physics Department, Faculty of Science, Beni-Suef University, Beni-Suef 62514, Egypt

[2] Department of Physics, Faculty of Science, Islamic University of Madinah, P. O. Box: 170, AlMadinah Almonawara 42351, Saudi Arabia

[3] Chemistry Department, Faculty of Science, Kuwait University, P.O. Box 5969, Safat 13060, Kuwait

[4] Polymer Research Laboratory, Chemistry Department, Faculty of Science, Beni-Suef University, Beni-Suef 62511, Egypt

* Correspondence: mssfadel@aucegypt.edu

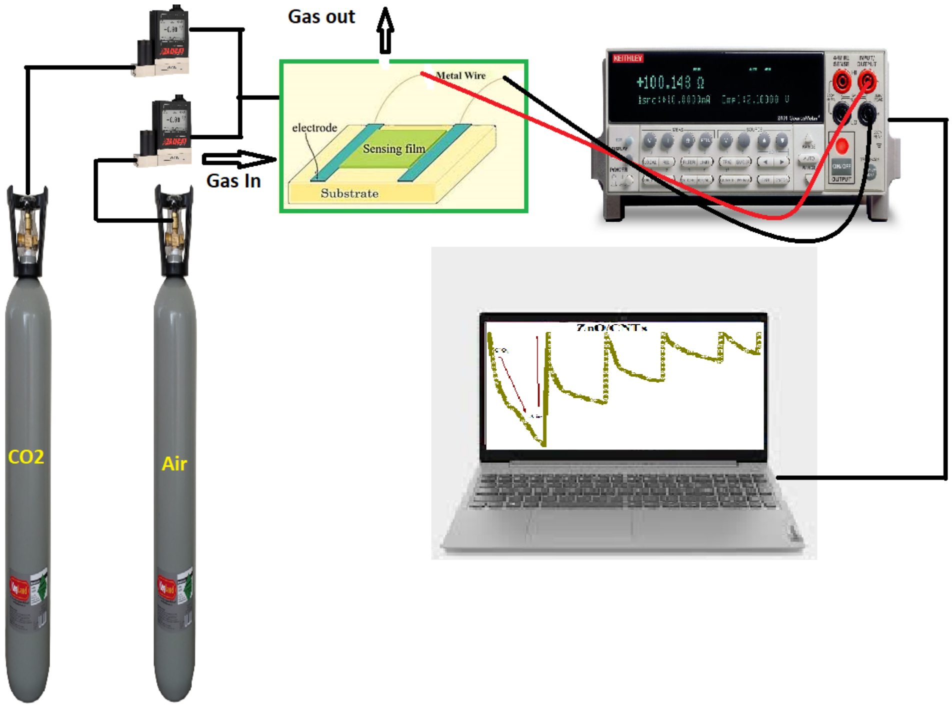

Figure S1. Schematic diagram for the homemade CO_2 gas sensor testing system.
